# Avoiding unnecessary biopsy in suspicious prostate cancer using PRIMARY score ≤3 and PSAD ≤0.2 ng/mL/cm^3^: a real-world, dual-center study from China

**DOI:** 10.1097/JS9.0000000000004733

**Published:** 2026-01-07

**Authors:** Jiawei He, Lin Qi, Minfeng Chen, Shuo Hu, Xiaomei Gao, Yongxiang Tang, Shouzhen Chen, Yi Cai

**Affiliations:** a Department of Urology, Disorders of Prostate Cancer Multidisciplinary Team, National Clinical Research Center for Geriatric Disorders, Xiangya Hospital, Central South University, Changsha, China; b Department of PET Center, National Clinical Research Center for Geriatric Disorders, Xiangya Hospital, Central South University, Changsha, China; c Department of Pathology, Disorders of Prostate Cancer Multidisciplinary Team, National Clinical Research Center for Geriatric Disorders, Xiangya Hospital, Central South University, Changsha, China; d Department of Urology, Qilu Hospital, Cheeloo College of Medicine, Shandong University, Jinan, China

**Keywords:** PI-RADS score, PRIMARY score, prostate cancer, prostate-specific antigen density

## Abstract

**Background:**

Among Chinese men with prostate-specific antigen (PSA) 4–20 ng/mL scheduled for biopsy, <20% harbor clinically significant prostate cancer (csPCa, International Society of Urological Pathology [ISUP] ≥2); most procedures target non-clinically significant prostate cancer (non-csPCa; ISUP <2) or benign prostatic hyperplasia (BPH). We tested whether prostate-specific membrane antigen (PSMA) positron-emission tomography/computed tomography (PET/CT) combined with multiparametric imaging and laboratory assays can accurately identify non-csPCa/BPH and safely reduce unnecessary biopsies.

**Materials and methods:**

We retrospectively enrolled patients from two centers. In the discovery cohort, participants underwent PSA testing, mpMRI, and ^68^Ga-PSMA-PET/CT, and were assessed for Prostate Imaging Reporting and Data System (PI-RADS) and PRIMARY scores. Receiver operating characteristic (ROC) curves, decision curve analysis, and diagnostic tests compared the efficacy of each indicator for non-csPCa/BPH and established the optimal strategy. The validation cohort independently verified this strategy.

**Results:**

Discovery cohort (*n* = 243): PRIMARY score area under the curve (AUC) 0.92 (95% confidence interval [CI]: 0.88–0.95); cutoff ≤3 provided 83.5% sensitivity and 90.9% specificity, outperforming PI-RADS (*P* = 0.002), PSA density (PSAD), free/total PSA (f/tPSA), and total PSA (tPSA) (all *P* < 0.0001). Combined models: PRIMARY score + PI-RADS AUC 0.95 (0.92–0.97); PRIMARY + PSAD or + f/tPSA both 0.94. A strategy of PRIMARY score ≤3 plus PSAD ≤0.2 achieved 100% specificity and positive predictive value (PPV) for csPCa, sparing 54.1% of unnecessary biopsies with zero csPCa missed – superior to European Association of Urology (EAU)-recommended PI-RADS ≤2 + PSAD ≤0.2 (sensitivity 19.6%). External cohort (*n* = 149) validated 100% specificity/PPV, avoiding 69.6% of biopsies while maintaining 0% csPCa miss rate.

**Conclusions:**

In men with suspected prostate cancer (PSA 4–20 ng/mL or abnormal digital rectal examination), the combined criterion of PRIMARY score ≤3 and PSAD ≤0.2 ng/mL/cm^3^ enabled pre-biopsy triage that safely avoided immediate diagnostic biopsy in more than 50% of patients ultimately found to harbor non-csPCa and BPH in our cohorts, while ensuring that no csPCa was missed. These findings require confirmation in larger prospective, multicenter studies before routine clinical implementation.

## Introduction

Biopsy and histopathological examination remain the gold standard for the definitive diagnosis of prostate cancer (PCa). In men with an initial suspicion of PCa due to elevated prostate-specific antigen (PSA) levels or abnormal digital rectal examination (DRE), current guidelines recommend multiparametric magnetic resonance imaging (mpMRI) as the initial imaging modality to determine the necessity of subsequent biopsy^[1]^, as it significantly improves the detection of clinically significant prostate cancer (csPCa)^[2,3]^. However, the high false-positive rate of mpMRI represents a critical limitation^[4–7]^; reliance on mpMRI alone results in approximately 42%–58% of suspected PCa cases referred for biopsy being ultimately confirmed as non-csPCa or benign prostatic hyperplasia (BPH) on final pathology^[8,9]^. Among Chinese men with PSA levels between 4 and 20 ng/mL who undergo biopsy, the positivity rate for csPCa is only 20%^[10]^, implying that roughly 5–8 out of every 10 patients undergoing guideline-recommended biopsy receive an unnecessary procedure. This constitutes a major clinical dilemma, necessitating the development of novel pre-biopsy strategies capable of identifying non-csPCa/BPH to refine biopsy decision-making.

Prostate-specific membrane antigen (PSMA)-targeted positron-emission tomography/computed tomography (PET/CT) molecular imaging has revolutionized the contemporary management of PCa^[11]^. PSMA PET/CT has previously demonstrated superior diagnostic accuracy to mpMRI for csPCa or intermediate- to high-risk PCa^[12–14]^. The pivotal question is whether PSMA PET/CT can safely obviate biopsy in patients with non-csPCa/BPH while avoiding missed diagnoses of csPCa. The PRIMARY score is a systematic PSMA PET/CT-based evaluation framework that integrates lesion anatomical location, imaging phenotype, and standardized uptake value (SUV) metrics; it has undergone real-world reproducibility and accuracy validation^[15]^ and has recently been incorporated into the Prostate Cancer Molecular Imaging Standardized Evaluation v2.0 criteria^[16,17]^.

The present study compared the diagnostic performance of distinct diagnostic pathway combinations based on the PRIMARY score, mpMRI, and PSA (including PSA-derived parameters) for identifying non-csPCa/BPH in biopsy-naïve men suspected of PCa on the basis of elevated PSA or abnormal DRE, with the objective of reducing unnecessary biopsies in patients ultimately proven to harbor non-csPCa/BPH.

Importantly, our focus is pre-biopsy triage: we evaluate whether PSMA PET/CT (PRIMARY score) combined with PSA density (PSAD) can improve decision-making at the point where a patient already meets guideline criteria for biopsy (e.g. PSA >4 ng/mL, abnormal DRE, or suspicious MRI), by identifying individuals who are very likely to have non-csPCa/BPH and for whom immediate biopsy might be safely deferred. This approach is distinct from active surveillance, which applies after a tissue diagnosis of low-risk disease.

## Materials and methods

### Study design

This work has been reported in line with the Standards for the Reporting of Diagnostic Accuracy studies criteria.

The discovery cohort of this retrospective study was based on patients from Xiangya Hospital, while the validation cohort was conducted at Qilu Hospital. The study protocol was approved by the Ethics Committee of both Hospitals. Research data were managed using the Electronic Data Capture system.

### Participants

We specifically included biopsy-naïve patients who met standard guideline indications for prostate biopsy (tPSA 4–20 ng/mL and/or abnormal DRE and/or suspicious mpMRI) and underwent PET/CT and mpMRI (discovery site). Our study therefore addresses the clinical decision point before biopsy – the pre-biopsy selection of patients who would otherwise be recommended for immediate diagnostic biopsy under current practice.

Patients in the discovery and validation cohorts were included if they had a tPSA level of 4–20 ng/mL and/or abnormal findings on DRE, clinical suspicion of PCa, and histopathological confirmation by biopsy. All patients were required to undergo mpMRI and PSMA PET/CT within 30 days prior to biopsy. Patients were excluded if they had missing imaging studies, exceeded the 30-day time window, underwent imaging after biopsy, or had a prior diagnosis and treatment.

**HIGHLIGHTS**

- In China, the clinically significant prostate cancer (csPCa) biopsy positivity rate is <20%, with many non-csPCa and benign prostatic hyperplasia (BPH) patients undergoing unnecessary biopsies.
- The PRIMARY score is a systematic evaluation scheme for prostate-specific membrane antigen positron-emission tomography/computed tomography, which is valuable for guiding biopsy decisions.
- The combined criterion PRIMARY score ≤3 plus PSA density ≤0.2 ng/mL/cm^3^ spares may allow pre-biopsy triage to safely avoid immediate diagnostic biopsy in a substantial proportion (>50%) of patients ultimately found to harbor non-csPCa/BPH, while maintaining no missed csPCa.

### mpMRI examination and image analysis

mpMRI was performed using a 3.0 T magnetic resonance scanner (Siemens, Germany) following a standardized protocol. The images were independently reviewed by two experienced radiologists in a blinded manner. The primary prostate lesions were assessed and scored according to the Prostate Imaging Reporting and Data System (PI-RADS) version 2.1^[18]^.

### PSMA PET/CT imaging and image analysis

The radiotracers ^68^Ga-PSMA-617^[19]^ (discovery cohort) and ^18^F-DCFPyL^[20]^ (validation cohort) were prepared as previously described. After intravenous injection, whole-body static PET/CT scans were performed using a PET/CT scanner (General Electric, USA) at least 60 minutes later. Two senior nuclear medicine physicians independently analyzed all images in a blinded manner. PET images and PET/CT fused images were reviewed in axial, coronal, and sagittal planes. Visual assessment was used to interpret the images, with any uptake higher than the background and not related to physiological uptake considered as a potential malignancy, and its maximum SUV (SUVmax) was measured. The PRIMARY score was assessed as previously described^[15]^. Specifically, a score of 1 indicates no dominant lesion or low-grade activity within the prostate. A score of 2 indicates diffuse central zone (TZ) activity or symmetrical peripheral zone (PZ) activity without extension to the prostate edge on CT. A score of 3 indicates focal TZ activity with visual intensity twice that of background TZ activity. A score of 4 indicates focal PZ activity (without a minimum intensity threshold). Any SUVmax value >12 is assigned a score of 5.

### Biopsy and histopathological examination

The biopsy protocol followed the previously described procedure^[21]^. After suspicious lesions were delineated on mpMRI by radiologists and on PET/CT by nuclear-medicine physicians, a urologist fused the preoperative targets to real-time transrectal ultrasound obtained with a BK Fusion platform and a biplanar 8848 probe. Each suspicious focus was sampled with 2–4 cores targeted to its volume. Targeted biopsies were completed first, after which a 12-core systematic transperineal template (eight PZ, three transition zone, one apex, fixed sequence, whole-gland coverage) was performed in the same position. The operator was blinded to imaging results and strictly adhered to the sequence PET/CT-targeted → mpMRI-targeted → systematic biopsy; if no suspicious lesion was identified on a given modality, the corresponding targeted step was omitted, and a systematic biopsy was performed directly. Specimens were independently reviewed by two senior genitourinary pathologists according to the International Society of Urological Pathology (ISUP) consensus^[22]^, and the highest grade was recorded as the final diagnosis: ISUP ≥2 was classified as csPCa, whereas ISUP <2 carcinomas and BPH were grouped as non-csPCa/BPH.

Among the 110 csPCa patients, 7.3% (8/110) were detected solely by systematic cores; 12.7% (14/110) exclusively by targeted sampling – mpMRI alone in 2.7% (3/110), PSMA PET/CT alone in 4.5% (5/110), and dual-imaging fusion in 5.5% (6/110) – while the remaining 80.0% (88/110) were diagnosed concordantly by both approaches, as detailed in Supplemental Digital Content Table available at: http://links.lww.com/JS9/G608.

### Statistical analysis

All statistical analyses were conducted using IBM SPSS Statistics, version 27.0.1 (IBM, Inc., Chicago, IL, USA) and R software, version 4.2.1 (R Foundation for Statistical Computing, Vienna, Austria). Quantitative data were tested for normality. Normally distributed data are presented as mean ± standard deviation (*x̄* ± *s*) and compared using independent samples *t*-tests. Non-normally distributed data are presented as median (interquartile range) and compared using the Mann–Whitney *U* test. Categorical data are presented as frequencies (percentages) and compared using the χ^2^ test. Areas under the curve (AUC) were compared using the DeLong test. Model clinical utility was assessed using calibration curves and decision curve analysis (DCA) based on the Dcurves package. Net benefit in DCA was defined as the number of true positives minus the weighted number of false positives (by threshold probability) divided by the total number of patients, with a threshold probability range of 0–0.40. Cohen’s Kappa coefficient was used to assess agreement between physicians on PRIMARY score and PI-RADS assessments. *P* value of <0.05 was considered statistically significant. Performance metrics included sensitivity, specificity, positive predictive value (PPV), and negative predictive value (NPV), all with 95% confidence intervals (CI). Optimal cutoff values were determined using the Youden index.

### Transparency in the reporting of artificial intelligence

This study has completed the TITAN Guideline Checklist^[23]^. No artificial intelligence tools were used for text or image generation and analysis.

## Results

### Baseline characteristics of the discovery cohort

The baseline characteristics of the 243 patients enrolled in the discovery cohort are summarized in Table 1. Non-csPCa/BPH accounted for 54.7% (133/243) of cases, of which BPH constituted 44.9% (109/243) and non-csPCa 9.9% (24/243); csPCa represented 45.3% (110/243). Across the entire cohort, the frequencies of PRIMARY scores 1–5 were 27.9%, 13.9%, 7.8%, 25.1%, and 25.1%, respectively; within the non-csPCa/BPH subgroup these proportions were 51.2%, 21.8%, 10.5%, 12%, and 4.5%. Significant differences were observed between non-csPCa/BPH and csPCa in terms of tPSA (7.630 *vs.* 11.81; *P* < 0.01), f/tPSA (0.15 *vs.* 0.11; *P* < 0.01), PSAD (0.16 *vs.* 0.32; *P* < 0.01), PI-RADS distribution (χ^2^ = 108.91; *P* < 0.01), and PRIMARY score distribution (χ^2^ = 141.44; *P* < 0.01).

**Table 1:** Overall characteristics and group comparison of the 243 patients in the discovery cohort.

Variable	Total (*n* = 243)	csPCa (*n* = 110)	Non-csPCa/BPH (*n* = 133)	Statistic	*P* value
Age,median (IQR), y	66.00 (61.00, 71.00)	66.50 (64.00, 71.00)	65.00 (59.00, 71.00)	*Z* = −1.73	0.08
BMI, median (IQR), kg/m^2^	23.63 (21.89, 25.04)	23.80 (21.54, 25.58)	23.50 (22.03, 24.60)	*Z* = −0.87	0.39
DRE, no. (%)				χ^2^ = 1.15	0.28
Normal	113 (46.50)	47 (42.73)	66 (49.62)		
Abnormal	130 (53.50)	63 (57.27)	67 (50.38)		
tPSA, median (IQR), ng/mL	9.73 (5.77, 14.06)	11.80 (8.25, 15.34)	7.63 (5.21, 12.13)	*Z* = −4.52	<0.01
f/tPSA, median (IQR), ng/mL	0.13 (0.09, 0.19)	0.11 (0.08, 0.15)	0.15 (0.12, 0.22)	*Z* = −5.33	<0.01
PSA density, median (IQR), ng/mL/cm^3^	0.22 (0.13, 0.39)	0.32 (0.21, 0.50)	0.16 (0.10, 0.27)	*Z* = −6.38	<0.01
SUVmax, median (IQR)	6.30 (0.00, 11.55)	11.55 (7.03, 17.30)	4.00 (0.00, 6.20)	*Z* = −10.21	<0.01
ISUP, no. (%)				χ^2^ = 243.00	<0.01
No cancer	109 (44.86)	0 (0.00)	109 (81.95)		
1	24 (9.88)	0 (0.00)	24 (18.05)		
2	41 (16.87)	41 (37.27)	0 (0.00)		
3	18 (7.41)	18 (16.36)	0 (0.00)		
4	18 (7.41)	18 (16.36)	0 (0.00)		
5	33 (13.58)	33 (30.00)	0 (0.00)		
PI-RADS, no. (%)				χ^2^ = 108.91	<0.01
1	13 (5.35)	2 (1.82)	11 (8.27)		
2	37 (15.23)	7 (6.36)	30 (22.56)		
3	108 (44.44)	24 (21.82)	84 (63.16)		
4	31 (12.76)	27 (24.55)	4 (3.01)		
5	54 (22.22)	50 (45.45)	4 (3.01)		
PRIMARY score, no. (%)				χ^2^ = 141.44	<0.01
1	68 (27.98)	0 (0.00)	68 (51.13)		
2	34 (13.99)	5 (4.55)	29 (21.80)		
3	19 (7.82)	5 (4.55)	14 (10.53)		
4	61 (25.10)	45 (40.91)	16 (12.03)		
5	61 (25.10)	55 (50.00)	6 (4.51)		

The Cohen’s κ coefficient for inter-reader agreement on the PRIMARY score was 0.80, and that for the PI-RADS score was 0.714.

BMI, body mass index; DRE, digital rectal examination; tPSA, total PSA; f/tPSA, free PSA/total PSA; PSAD, PSA density; PI-RADS, Prostate Imaging Reporting and Data System; ISUP, International Society of Urological Pathology; Z, Mann–Whitney test, χ^2^, Chi-square test.

### PRIMARY score significantly enhances diagnostic performance for non-csPCa/BPH

**Figure 1.**
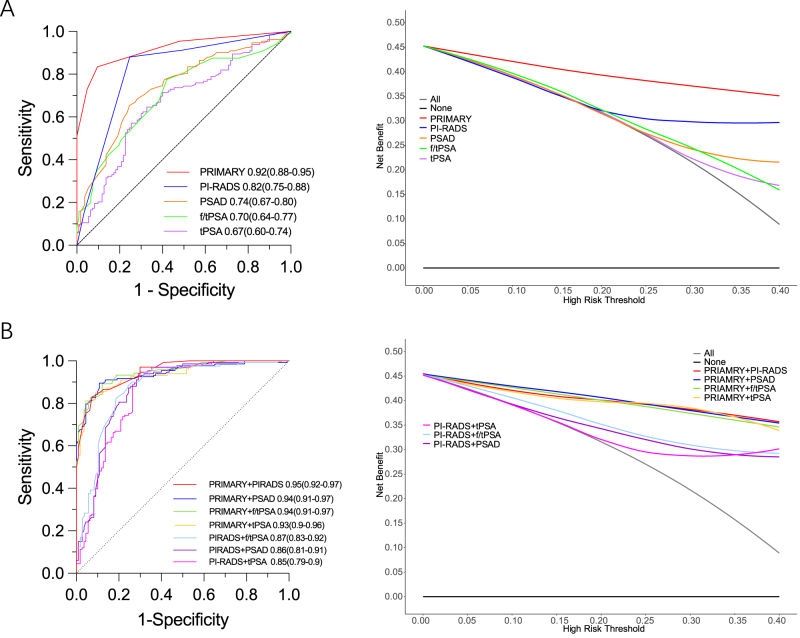
ROC (left) and decision-curve analysis (right) for predicting non-clinically significant prostate cancer or benign prostatic hyperplasia (non-csPCa/BPH) in the discovery cohort (*n* = 243). (A) Single tests. (B) Combined models. AUC (95% CI) and net benefit across 0%–40% threshold probabilities are shown in the legends.

ROC curves were constructed, and AUC values were calculated based on the differential clinical features between non-csPCa/BPH and csPCa to assess the diagnostic performance of various tests for non-csPCa/BPH. Among single tests, the AUC for PRIMARY score was 0.92 (95% CI: 0.88–0.95), significantly higher than PI-RADS (*P* = 0.002), PSAD (*P* < 0.0001), f/tPSA (*P* < 0.0001), and tPSA (*P* < 0.0001). DCA showed that PRIMARY score had the highest net benefit in the 5%–40% threshold interval, indicating its superior performance in distinguishing non-csPCa/BPH. In contrast, lower AUC and DCA values for tPSA (0.67) and f/tPSA (0.7) corresponded to poorer diagnostic ability (Fig. 1A). Analysis of combined diagnostic models showed that incorporating PRIMARY score significantly improved diagnostic performance (*P* < 0.01), with PRIMARY score + PI-RADS yielding the best performance, an AUC of 0.95 (95% CI: 0.92–0.97), followed by PRIMARY score + PSAD (AUC = 0.94, 95% CI: 0.91–0.97) and PRIMARY score + f/tPSA (AUC = 0.94, 95% CI: 0.91–0.97). The diagnostic performance of PRIMARY score combinations (AUC = 0.93–0.94) was higher than that of PI-RADS combinations (AUC = 0.85–0.87). Goodness-of-fit testing for the models is presented in Supplemental Digital Content Figure 1A and B available at: http://links.lww.com/JS9/G607. DCA further demonstrated that PRIMARY score combinations had higher net benefit in the 5%–40% threshold interval (Fig. 1B). These results highlight the key role of PRIMARY score in both single and combined tests.

Our ultimate goal was to completely avoid biopsy in non-csPCa/BPH patients, thus focusing on the specificity and PPV of all models, which represent the ability to accurately identify non-csPCa/BPH. Diagnostic test results showed that among single tests, PI-RADS had the highest sensitivity (93.99%, 95% CI: 88.11–97.18), but specificity was only 70.60% (95% CI: 61.41–78.17), with a false-positive rate as high as 20.88% (95% CI: 15.00–28.22), which may lead to overtreatment through excessive biopsies; the optimal cutoff value for PI-RADS was 3. In contrast, PRIMARY score (optimal cutoff value of 3) showed a more balanced specificity of 90.91% (95% CI: 83.52–95.31), sensitivity (83.46%, 95% CI: 75.80–89.13), and a lower false-positive rate of 8.26% (95% CI: 4.25–15.05). Combined diagnostic results showed that adding PRIMARY score increased the specificity and PPV of the tests compared to single tests, indicating improved accuracy in identifying non-csPCa/BPH. PRIMARY score combined with PI-RADS had excellent performance, with sensitivity of 78.19% (95% CI: 70.03–84.69) and specificity and PPV of 94.55% (95% CI: 88.02–97.76).

A key finding was that the combination of PRIMARY score≤3 and PSAD ≤0.2 achieved 100% specificity and PPV, outperforming all other single or combined models and conferring a unique advantage in confirming non-csPCa/BPH. Under this criterion, 54.14% of patients would be spared biopsy completely, without missing any csPCa (specificity 100% [95% CI: 95.79–100]). By contrast, the European Association of Urology (EAU) guideline-recommended biopsy-avoidance strategy combining PI-RADS ≤2 with PSAD ≤0.2 exhibited only 19.55% sensitivity (95% CI: 13.39–27.51) in our discovery cohort and entailed a 1.82% risk of csPCa under-detection (specificity 98.18% [95% CI: 92.94–99.68]). These data underscore PRIMARY score ≤3 plus PSAD ≤0.2 as the optimal strategy for confirming non-csPCa/BPH (Table 2).

**Table 2:** Diagnostic performance of single and combined tests for non-csPCa/BPH.

Strategy (cutoff value)	Sensitivity	Specificity	PPV	NPV
PRIMARY (≤3)	83.46 (75.8–89.13)	90.91 (83.52–95.31)	91.74 (84.95–95.75)	81.97 (73.75–88.12)
PI-RADS (≤3)	93.99 (88.11–97.18)	70 (60.41–78.17)	79.11 (71.78–84.99)	90.59 (81.79–95.56)
PSAD (≤0.2)	65.41 (56.61–73.31)	76.36 (67.13–83.71)	76.99 (67.94–84.16)	64.61 (55.69–72.66)
f/tPSA (≥0.115)	69.92 (61.27–77.40)	59.09 (49.29–68.24)	67.39 (58.81–74.98)	61.9 (51.87–71.05)
tPSA (≤9.74)	64.66 (55.85–72.62)	67.27 (57.58–75.74)	70.49 (61.45–78.23)	61.16 (51.84–69.75)
PRIMARY (≤3) + tPSA (≤9.74)	52.63 (43.82–61.29)	98.18 (92.94–99.68)	97.22 (89.42–99.52)	63.16 (55.41–70.29)
PRIMARY (≤3) + f/tPSA (≥0.115)	58.65 (49.77–67.01)	94.55 (88.02–97.76)	92.86 (84.53–97.06)	65.41 (57.41–72.65)
PRIMARY (≤3) + PSAD (≤0.2)	54.14 (45.29–62.73)	100 (95.79–100)	100 (93.69–100)	64.33 (56.61–71.39)
PI-RADS (≤3) + tPSA (≤9.74)	58.65 (49.77–67.01)	85.45 (77.15–91.2)	82.98 (73.53–89.66)	63.09 (54.76–70.73)
PI-RADS (≤3) + f/tPSA (≥0.115)	66.17 (57.39–73.99)	87.27 (79.24–92.61)	86.27 (77.71–92.02)	68.09 (59.63–75.54)
PI-RADS (≤3) + PSAD (≤0.2)	62.41 (53.55–70.53)	90.91 (83.52–95.31)	89.25 (80.69–94.44)	66.67 (58.45–74.02)
PRIMARY (≤3) + PI-RADS (≤3)	78.19 (70.03–84.69)	94.55 (88.02–97.76)	94.55 (88.02–97.76)	78.19 (70.03–84.69)
PI-RADS (≤2) + PSAD (≤0.2)^a^	19.55 (13.39–27.51)	98.18 (92.94–99.68)	92.86 (75.04–98.75)	50.23 (43.38–57.08)

NPV, negative predictive value; PPV, positive predictive value.

^a^ The EAU guidelines recommend a strategy of omitting biopsy and offering PSA monitoring.

### External validation cohort

To further validate the efficacy of the combination of PRIMARY score and PSAD for non-csPCa/BPH, we conducted a retrospective analysis in another medical center (Qilu Hospital). The external validation cohort comprised 149 patients who underwent ^18^F-DCFPyL PET/CT before biopsy. Non-csPCa/BPH accounted for 61.7% (92/149) of cases, with BPH constituting 55% (82/149) and non-csPCa 6.7% (10/149); csPCa represented 38.3% (57/149). The characteristics of the patients included in the validation cohort are shown in Table 3.

**Table 3:** Comparison of characteristics between non-csPCa/BPH and csPCa in the validation cohort.

Variable	Total (*n* = 149)	csPCa (*n* = 57)	Non-csPCa/BPH (*n* = 92)	Statistic	*P* value
Age, median (IQR), y	67.00 (62.00, 73.00)	68.00 (63.00, 73.00)	67.00 (61.75, 72.00)	*Z* = −1.16	0.25
tPSA, median (IQR), ng/mL	6.27 (4.08, 8.60)	8.60 (6.05, 12.80)	5.08 (3.49, 6.92)	*Z* = −6.21	<0.01
PSA density, median (IQR), ng/mL/cm^3^	0.18 (0.10, 0.31)	0.32 (0.20, 0.43)	0.13 (0.07, 0.19)	*Z* = −7.44	<0.01
SUVmax, median (IQR)	6.40 (3.60, 11.40)	15.20 (8.30, 24.90)	4.60 (2.98, 6.62)	*Z* = −7.87	<0.01
ISUP, no. (%)					
No cancer	82 (55.03)	0 (0.00)	82 (89.13)	–	<.01
1	10 (6.71)	0 (0.00)	10 (10.87)		
2	12 (8.05)	12 (21.05)	0 (0.00)		
3	10 (6.71)	10 (17.54)	0 (0.00)		
4	14 (9.40)	14 (24.56)	0 (0.00)		
5	21 (14.09)	21 (36.84)	0 (0.00)		
PRIMARY score, no. (%)				χ^2^ = 105.08	<0.01
1	20 (13.42)	0 (0.00)	20 (21.74)		
2	51 (34.23)	1 (1.75)	50 (54.35)		
3	14 (9.40)	2 (3.51)	12 (13.04)		
4	29 (19.46)	21 (36.84)	8 (8.70)		
5	35 (23.49)	33 (57.89)	2 (2.17)		

Z, Mann–Whitney test, χ^2^, Chi-square test, –: Fisher exact.

As illustrated in Table 4, the external validation cohort confirmed that PRIMARY score maintained high specificity (94.74%, 95% CI: 84.45–98.63) and PPV (96.47%, 95% CI: 89.32–99.08) for non-csPCa/BPH, while preserving balanced sensitivity (89.13%, 95% CI: 80.49–94.38) and NPV (84.38%, 95% CI: 72.68–91.86). Moreover, the composite criterion PRIMARY score ≤3 + PSAD ≤0.2 yielded a sensitivity of 69.57% (95% CI: 58.97–78.5), with specificity and PPV both reaching 100% (95% CI: 92.13–100 and 92.95–100, respectively). These data indicate that, in the external cohort, 69.57% of patients with non-csPCa/BPH could safely avoid biopsy without missing any csPCa. Collectively, the findings validate the combination of PRIMARY score + PSAD as an excellent strategy for reducing unnecessary biopsies in non-csPCa/BPH.

**Table 4:** Diagnostic performance of single and combined tests for non-csPCa/BPH in the validation cohort.

Strategy (cutoff value)	Sensitivity	Specificity	PPV	NPV
PRIMARY (≤3)	89.13 (80.49–94.38)	94.74 (84.45–98.63)	96.47 (89.32–99.08)	84.38 (72.68–91.86)
PSAD (≤0.2)	76.09 (65.85–84.1)	75.44 (61.96–85.47)	83.33 (73.27–90.27)	66.15 (53.26–77.13)
PRIMARY (≤3) + PSAD (≤0.2)	69.57 (58.97–78.5)	100 (92.13–100)	100 (92.95–100)	67.06 (55.92–76.64)

NPV, negative predictive value; PPV, positive predictive value.

## Discussion

In men with suspected PCa who have not yet undergone biopsy, the long-standing debate has centered on maximally reducing unnecessary biopsies for non-csPCa and benign disease without missing csPCa^[24]^. Multiple strategies have been developed to improve csPCa detection, including mpMRI; however, the problem of over-biopsy persists. The GÖTEBORG-2 study biopsied men with PSA >3 ng/mL and found a csPCa prevalence of only 27.4%^[25]^; similarly, the PROMIS study, using a PSA threshold >15 ng/mL, reported a csPCa rate of approximately 53.6%^[8]^, whereas the rate in China is as low as 20%. Thus, a substantial proportion of non-csPCa and benign conditions undergo unnecessary biopsy. Accurate identification of these individuals before biopsy is therefore an effective means of averting over-diagnosis. The PRECISION trial demonstrated that the MRI pathway (MRI with or without MRI-targeted biopsy) outperforms systematic biopsy in detecting ISUP grade 2 or higher cancer among patients with PI-RADS 3–5. Patients with PI-RADS 1–2 are spared from biopsy. However, only 38% of csPCa cases were detected among those undergoing MRI-targeted biopsy^[26,27]^. The equivocal PI-RADS = 3 group and the high false-positive rate of MRI limit its effectiveness in covering non-csPCa/BPH patients. Accurate pre-biopsy identification of this population is crucial to avoid overdiagnosis, thus necessitating the development of new diagnostic strategies^[28]^.

PSMA PET/CT has redefined the diagnostic algorithm for PCa^[29]^. ^68^Ga-PSMA PET/CT is supported by high-level evidence^[14]^and is increasingly employed for staging and biochemical-recurrence assessment; nevertheless, its use in the primary diagnostic setting is still considered experimental^[30–32]^. Using post-hoc analysis of the prospective PRIMARY trial, Emmett et al. constructed a systematic scoring framework to enhance the accuracy and robustness of PSMA PET/CT interpretation^[15,33]^. By integrating pattern (focal vs diffuse), zonal location and high SUVmax, the PRIMARY score demonstrates high precision for csPCa detection. Reliance on pattern rather than uptake intensity mitigates variability in PSMA expression encountered in benign disease and low-grade tumours, conferring particular utility in distinguishing BPH, prostatic intraepithelial neoplasia (PIN), and ISUP grade 1 PCa during screening. Reduced dependence on SUVmax also promotes generalisability and consistency across different PET cameras and PSMA ligands, with minimal anticipated variability in pattern attribution^[17]^. In this study, the AUC of ^18^F-DCFPyL (0.955) was higher than that of ^68^Ga-PSMA-617 (0.918), but the difference did not reach statistical significance (ΔAUC = 0.037; 95% CI – 0.012–0.086; *P* = 0.13, DeLong test). Patient-based analysis showed that the PRIMARY scores of both tracers were effective in distinguishing csPCa from non-csPCa/BPH patients. Inter-rater agreement assessed by Cohen’s κ was 0.80 for PRIMARY versus 0.714 for PI-RADS, consistent with prior observation^[15,34]^.

In this study, we constructed and compared multiple models with the explicit endpoint of detecting non-csPCa/BPH, emphasising specificity and PPV as metrics that quantify the accurate identification of non-csPCa/BPH. To our knowledge, this is the first investigation specifically aimed at averting unnecessary biopsy in patients whose final histopathology confirms non-csPCa/BPH. We note a frequent source of misunderstanding: active surveillance is a management strategy for patients already diagnosed with low-risk PCa, whereas our proposed PRIMARY + PSAD approach is intended as pre-biopsy triage. That is, it aims to reduce unnecessary immediate diagnostic biopsies among patients who currently meet biopsy criteria but have a high probability of benign disease or non-csPCa on final pathology. It is not intended to replace tissue diagnosis when indicated, nor to alter post-diagnosis management pathways such as active surveillance.

The first key finding was that the PRIMARY score delivered the most balanced performance among all single-modal assessments. Comparative ROC and decision-curve analyses demonstrated that PRIMARY score conferred a superior increment in specificity relative to PI-RADS. Moreover, the combination PRIMARY score ≤3 + PI-RADS ≤3 achieved the highest AUC (0.95, 95% CI: 0.92–0.97), aligning with prior reports that integrating PRIMARY score with MRI improves NPV and sensitivity for csPCa^[35,36]^.

A second critical observation was that PRIMARY score ≤ 3 + PSAD ≤ 0.2 attained 100% specificity (95% CI: 95.79–100%) and 100% PPV (95% CI: 93.69–100%), conferring unmatched reliability for the confirmation of non-csPCa/BPH. Decision-curve analysis revealed the highest net benefit within the 5%–25% threshold range; application of this rule accurately classified 54.14% of non-csPCa/BPH cases without missing any csPCa (specificity 100% [95% CI: 95.79%–100%]). The 2025 EAU guidelines endorse PI-RADS ≤ 2 + PSAD ≤ 0.2 as a valid biopsy-avoidance strategy. Exploratory analysis of this rule in our discovery cohort showed a low non-csPCa/BPH detection rate (sensitivity 19.55%, 95% CI: 13.39–27.51) and a 1.82% csPCa under-detection rate (specificity 98.18%, 95% CI: 92.94–99.68), underscoring the need for an alternative approach. Beyond the discovery cohort, we externally validated the PRIMARY score ≤ 3 + PSAD ≤ 0.2 strategy in an additional 149 patients from a separate tertiary center. The combination again correctly identified approximately 70% of non-csPCa/BPH cases without missing csPCa, mirroring the discovery-cohort findings. Given that the two institutions serve distinct geographical regions of China, the concordant results strongly support the generalisability of this combined diagnostic strategy. The 2025 EAU guidelines endorse PI-RADS ≤2 + PSAD ≤0.2 as a valid biopsy-avoidance strategy. Exploratory analysis of this rule in our discovery cohort showed a low non-csPCa/BPH detection rate (sensitivity 19.55%, 95% CI: 13.39–27.51) and a 1.82% csPCa under-detection rate (specificity 98.18%, 95% CI: 92.94–99.68), underscoring the need for an alternative approach. Beyond the discovery cohort, we externally validated the PRIMARY score ≤ 3 + PSAD ≤ 0.2 strategy in an additional 149 patients from a separate tertiary center. The combination again correctly identified approximately 70% of non-csPCa/BPH cases without missing csPCa, mirroring the discovery-cohort findings. Given that these two institutions serve distinct geographical regions of China, the concordant results strongly support the generalisability of this combined diagnostic strategy.

In this study, a novel biopsy-decision algorithm was established for a well-defined cohort of patients with clinically suspected PCa, enabling a substantial reduction in unnecessary biopsies and associated morbidity in individuals ultimately proven to harbor non-csPCa/BPH. PRIMARY score demonstrated superior biopsy-guiding value for the non-csPCa/BPH population compared with PI-RADS. When PSMA PET/CT is performed directly after baseline blood tests – bypassing mpMRI – more than half of patients with non-csPCa/BPH are accurately spared biopsy without any csPCa being missed. If mpMRI has already been completed, the subsequent addition of PSMA PET/CT further improves the detection of non-csPCa/BPH.

Additionally, using current Chinese medical-service tariffs, we conducted a deterministic cost-effectiveness analysis across all 392 study patients. Compared with the standard “PSA + mpMRI” pathway, the stand-alone “PSA + PET/CT” strategy reduced total costs approximately by ¥376,400 (an 11.7% decrease) while avoiding 166 unnecessary invasive biopsies, yielding an average saving of ¥1 573 per avoided procedure – demonstrating both economic and procedural advantages. In contrast, adding PSMA PET/CT to mpMRI (the triple strategy) achieved a similar reduction in unnecessary biopsies but increased total expenditure by ¥211 600, corresponding to an incremental cost of ¥1324 per patient. Therefore, the combined approach may be justified only in centers prioritizing maximal diagnostic certainty regardless of cost.

This study has limitations. Despite being multicentric, the sample size was still insufficient, and selection bias was inevitable. Further validation with large-scale external data is needed. Although PRIMARY score has been evaluated for reproducibility, interpretation may vary across institutions and physicians, leading to inconsistent classifications. Given the absence of direct head-to-head comparative evidence for the two tracers, the conclusions drawn from this study are primarily applicable to the specific clinical contexts in which each tracer was utilized within their respective cohorts. More robust validation of consistency will require the completion of future prospective crossover trials. Additionally, given that not all patients underwent radical prostatectomy, the biopsy tissues we obtained may not fully represent the true nature of the tumor. Finally, further work is needed to assess the cost-effectiveness of this combined approach.

## Conclusions

In men with suspected prostate cancer (PSA 4–20 ng/mL or abnormal DRE), the combined criterion of PRIMARY score ≤3 and PSAD ≤0.2 ng/mL/cm^3^ enabled pre-biopsy triage that safely avoided immediate diagnostic biopsy in more than 50% of patients ultimately found to harbor non-csPCa and BPH in our cohorts, while ensuring that no csPCa was missed. These findings require confirmation in larger prospective, multicenter studies before routine clinical implementation.

## Data Availability

Due to patient privacy protections at the two participating institutions, the datasets generated and/or analyzed during the current study are not publicly available; they may be obtained from the corresponding author upon reasonable request.

## Research registration unique identifying number (UIN)

The clinical research was pre-registered (ClinicalTrials.gov ID: NCT05073653) https://www.clinicaltrials.gov/study/NCT05073653?cond=NCT05073653&rank=1.
